# Fungal Secondary Metabolites in Bioelectrochemical Systems: A Bibliometric Analysis and Critical Review of Emerging Trends and Challenges for Sustainable Energy

**DOI:** 10.3390/molecules31040716

**Published:** 2026-02-19

**Authors:** Segundo J. Rojas-Flores, Rafael Liza, Renny Nazario-Naveda, Félix Díaz, Daniel Delfin-Narciso, Moisés Gallozzo Cardenas

**Affiliations:** 1 Facultad de Ingeniería y Arquitectura, Universidad Autónoma del Perú, Lima 15842, Peru; felix.diaz@autonoma.pe; 2 Escuela de Posgrado, Universidad Continental, Lima 15113, Peru; rlizan@continental.edu.pe; 3 Departamento de Ciencias, Universidad Tecnológica del Perú, Trujillo 13011, Peruc21228@utp.edu.pe (M.G.C.); 4 Grupo de Investigación en Ciencias Aplicadas y Nuevas Tecnologías, Universidad Privada del Norte, Trujillo 13011, Peru; daniel.delfin@upn.edu.pe

**Keywords:** fungal secondary metabolites, bioelectrochemistry, sustainable energy, redox mediators, sustainability

## Abstract

The global energy crisis driven by an 80% reliance on fossil fuels and the urgent need to reduce greenhouse gas emissions demands the exploration of sustainable biotechnological alternatives. This study addresses a critical knowledge gap regarding the integration of fungal secondary metabolites into bioelectrochemical energy systems, as these compounds have traditionally been investigated for pharmacological purposes. The methodology involved a documentary analysis using the Scopus database (2000–2025), applying a search equation that combined terms such as “secondary metabolite”, “fungi”, and “bioenergy”. Data processing was conducted using R Studio (R 3.6.0+), VOSviewer (1.6.20) for collaboration networks, and Plotly Studio (v6.5.0) for interactive visualizations. Key findings revealed that redox mediators such as quinones and organic acids derived from *Aspergillus niger* enhanced electron transfer efficiency by 35%, achieving power densities of 1.2 W/m^2^. Meanwhile, *Penicillium chrysogenum* reduced internal electrode resistance by 40%. Additionally, the “*xeno-fungosphere*” system achieved 97.9% herbicide removal and generated 9.3 µW/cm^2^. Notably, biosynthesized bis-quinones were successfully applied in redox flow batteries, reaching a capacity of 1.58 Ah/L. In conclusion, the study identified a scientific shift from pharmacological applications toward energy metabolism and sustainability, positioning fungi as critical components for the future efficiency of bioelectrical technologies.

## 1. Introduction

Current energy challenges reflect a global crisis marked by the sustained increase in demand and the dependence on fossil sources that generate severe environmental impacts [1]. According to the International Energy Agency, in 2024, global primary energy consumption reached 604 exajoules, of which more than 80% came from fossil fuels, responsible for approximately 73% of greenhouse gas emissions [2,3]. This situation has been exacerbated by the growth of the world population, which surpassed 8 billion inhabitants in 2023, and projections indicate that by 2050, energy demand will increase by 50% [4]. In parallel, air pollution resulting from coal and oil combustion causes more than 7 million premature deaths annually, according to the World Health Organization [5]. These data highlight the urgency of exploring sustainable alternatives that reduce dependence on non-renewable resources and promote clean technologies [6]. In this context, fungal secondary metabolites have emerged as an innovative source for bioelectrochemical applications, capable of transforming biological processes into usable energy, offering a bridge between biotechnology and energy sustainability [7].

The relevance of fungal secondary metabolites in bioelectrochemistry lies in their ability to act as redox mediators, facilitating electron transfer between microorganisms and electrodes [8]. Compounds such as menadione (C_11_H_8_O_2_), aromatic quinones (C6H4O2), and phenolic derivatives like gallic acid (C_7_H_6_O_5_) have demonstrated efficiency in generating electrical current through oxidation–reduction reactions [9,10]. Currently, solutions have been developed that integrate metabolite-producing fungi with modified electrodes, achieving improvements in power density and system stability [11]. Applications are also being explored in wastewater treatment, where fungal metabolites contribute not only to energy generation but also to contaminant degradation [12]. These approaches represent significant advances toward energy transition, as they combine the valorization of biological resources with the production of sustainable energy, offering viable alternatives to conventional systems [13].

One study demonstrated that metabolites derived from *Aspergillus niger* increased electron transfer efficiency in microbial fuel cells by 35%, reaching a power density of 1.2 W/m^2^, evidencing the capacity of fungi to enhance bioelectrochemical systems [14]. Reports also highlight the use of metabolites from *Penicillium chrysogenum* as redox mediators, achieving a 40% reduction in internal electrode resistance and improved operational stability over 60 continuous days, consolidating the viability of these compounds in long-term applications [15,16]. Likewise, collaborative projects in Europe and Asia have shown that secondary metabolites from marine fungal species can be integrated into hybrid bioelectrochemical and mechanical storage systems, achieving conversion efficiencies above 50% and opening new perspectives for the integration of biotechnology into smart energy grids [17,18]. These results confirm that recent advances not only validate the potential of fungal metabolites but also position bioelectrochemistry as a strategic field for global sustainability.

Conducting a bibliometric analysis on this topic is essential to understand the evolution of knowledge and research trends. The Scopus database serves as a key resource for obtaining reliable and updated information on scientific publications, enabling the identification of leading authors, institutions, and countries in the field [19]. Tools such as R Studio facilitate statistical data processing, while VOSviewer enables the construction of collaboration and co-occurrence maps, revealing the most influential research networks [20]. Plotly, in turn, provides interactive visualizations that enrich result interpretation and enhance scientific communication [21]. This methodological approach not only adds rigor to the study but also helps identify knowledge gaps and strategic opportunities for future research, consolidating the relevance of the topic in academic and technological contexts [22]. The main knowledge gap lies in the limited integration of fungal secondary metabolites into bioelectrochemical systems applied to sustainable energy, as most studies remain focused on medical or pharmacological applications, leaving their energetic and environmental potential unexplored. The choice of fungal secondary metabolites as the central focus of this study, rather than other types of metabolites (such as primary ones) or synthetic mediators, is justified by several fundamental reasons. Primary metabolites (such as sugars or amino acids) are essential for cellular growth, but they generally lack the structural specificity and redox potential required for efficient and sustained electron mediation in bioelectrochemical systems. Synthetic mediators, on the other hand, such as potassium ferricyanide or certain dyes, while effective, can be toxic, costly to synthesize, and nonrenewable. In contrast, fungal secondary metabolites, including quinones (e.g., *anthraquinone fungiquinone*), organic acids (such as oxalic acid produced by *Aspergillus niger*), and lactones, offer a unique combination of properties: they possess intrinsically redox-active functional groups within their structures, are sustainably produced through fermentation processes using low-cost residual biomass, and exhibit vast chemical diversity shaped by millions of years of evolution. This diversity represents an untapped reservoir of novel molecules with electron-transfer capabilities, biocompatibility, and biodegradability, positioning them as a superior and more environmentally friendly alternative to drive the transition toward sustainable energy technologies.

The primary objective of this study was to analyze the potential of fungal secondary metabolites in bioelectrochemical applications for sustainable energy, identifying their benefits, limitations, and future perspectives. Secondary objectives include: (Q1) identifying key fungal metabolites with redox capacity; (Q2) evaluating their performance in recent bioelectrochemical systems; (Q3) analyzing complementary technologies that enhance their application; (Q4) determining international research trends and collaboration; and (Q5) proposing action lines for integrating these compounds into energy transition strategies. Research in this area is crucial, as it unlocks a sustainable and diverse source of redox biocatalysts. Advancing this field can overcome technical limitations in bioelectrochemistry, driving the development of efficient biobatteries, robust biosensors, and advanced bioremediation strategies, ultimately integrating biology and technology for a cleaner energy future.

## 2. Results and Analysis

Table 1 presents a selection of ten scientific articles that have had a significant impact on the study of metabolites with redox properties. The most cited article was by Huang et al. (2008) [23], published in Environmental Science and Technology. This work addressed both lignin derivatives and fungal metabolites, reflecting its multidisciplinary relevance. The study focused on the degradation of lead-contaminated lignocellulosic waste by the fungus *Phanerochaete chrysosporium*, highlighting the role of aromatic polymers with multiple redox sites and fungal secondary metabolites with redox capacity. Its high citation count underscores its influence in the fields of environmental biotechnology and sustainable chemistry. The article by Hasler et al. (1997) [24] examined the determination of psilocin and 4-hydroxyindole-3-acetic acid in human plasma, positioning organic acids as key metabolites in pharmacokinetics and neurochemistry. Despite its age, this study remains highly relevant, indicating the enduring importance of redox compounds in biomedical contexts. The third most cited article, with 172 mentions, was by Vesth et al. (2018) [25], published in Nature Genetics, which investigated genomic variation in species of the genus *Aspergillus*, emphasizing the role of organic acids in fungal metabolic diversity. This work demonstrates how genomics has revitalized the study of redox metabolites by integrating sequencing tools with functional analysis.

Following this, the study by Berthelot et al. (2016) [26] examined dark endophytes tolerant to metals in contaminated sites, reinforcing interest in organic acids as agents of plant promotion and bioremediation. In the field of flavonoids, the article by Fouda et al. (2023) [27] has already reached 111 citations, which is remarkable for such a recent publication. This work on the green synthesis of zinc oxide nanoparticles using Punica granatum extract highlighted the application of polyphenolic compounds in catalysis and antimicrobial activity, evidencing the rise of green nanotechnology. The article by Loi et al. (2021) [28] revisited the role of lignin derivatives in sustainability, while the study by Debnath et al. (2008) [29], reaffirmed the value of flavonoids in stress-resistant plants. Also, the work of Corduneanu et al. (2006) [30] on the electrochemical oxidation of resveratrol contributed to understanding polyphenols as redox-active antioxidants. The articles by Ullah et al. (2013) [31] and Oliveira et al. (2007) [32], with 64 and 60 citations respectively, addressed yeast adaptation to weak acids and the oxidation of ochratoxin A, showing how organic acids and fungal metabolites are linked to energy efficiency and environmental toxicology. Taken together, the table reveals that lignin derivatives and organic acids are the most recurrent and cited metabolites, suggesting their centrality in bioremediation, sustainability, and microbial metabolism. Furthermore, the diversity of sources from microbiology to genetics and nanotechnology journals reflects the cross-disciplinary nature of redox compound research across multiple scientific domains. This bibliometric overview not only identifies the most influential studies but also illustrates how the redox properties of natural metabolites have been harnessed in environmental, biomedical, and technological contexts over time.

Table 2 presents a diverse and highly cited landscape of research that, while not directly focused on fungal secondary metabolites for energy applications, provides essential technological context and highlights areas of potential synergy. The analysis of these studies highlights the maturity and impact of bioelectrochemical technologies in adjacent fields such as biosensing and bioremediation domains that could directly benefit from the innovative integration of fungal compounds. A dominant theme is the application of bioelectrochemical platforms in analytical detection (biosensors), with the two most cited articles in the table (396 and 212 citations, respectively) dedicated to photoelectrochemical immunoassays for mycotoxins [33,34]. These studies, which utilize materials such as TiO_2_ and CdTe quantum dots, demonstrate the high sensitivity and specificity achievable with advanced bioelectrochemical systems. Their relevance is twofold: mycotoxins are themselves fungal secondary metabolites with significant implications for food safety, establishing a direct link between the fungal kingdom and cutting-edge electrochemical applications. Furthermore, the architecture of these biosensors, often requiring redox mediators or biocatalytic layers for signal transduction, offers an ideal opportunity to replace synthetic components with redox-active fungal metabolites (e.g., quinones or pigments), potentially leading to more biocompatible, stable, and cost-effective devices.

Additionally, the field of bioremediation and wastewater treatment emerged as another key area of application, supported by highly cited reviews. The work by Langbehn et al. on the biological removal of antibiotics [35] emphasized the urgency and complexity of addressing effluent contamination. Bioelectrochemical systems, such as microbial fuel cells, are a promising technology in this domain [36,37,38]. The integration of fungal metabolites could enhance these systems by acting as potent electron mediators for the degradation of recalcitrant pollutants, such as azo dyes studied by de Almeida et al. [39], or even by inhibiting microbially induced corrosion, a phenomenon investigated by Zhang et al. in aluminum alloys [38]. This latter study is particularly illustrative of the dual nature of fungal interactions: while fungi like *Aspergillus niger* can promote corrosion, their metabolites (or antifungals such as miconazole nitrate used in the study) can be employed to control it, opening a new research avenue for the use of fungal extracts as corrosion inhibitors in bioelectrochemical infrastructure. The table includes more fundamental investigations addressing relevant transport and toxicity mechanisms. The article on ABC transporters in yeast [37] and the review on forensic toxicology of higher fungi [40] highlighted the complexity of fungal biology and the potent bioactivity of their metabolites [41,42]. Understanding these mechanisms such as the role of transporters in compound secretion is essential for designing effective production and application strategies for metabolites in bioelectrochemical systems. For instance, the ability of certain metabolites to chelate metals or facilitate electron transport could be harnessed in the development of bioconductors or catalytic interfaces.

Table 3 reveals a high-impact technological convergence, where bioactive fungal compounds are integrated with bioelectrochemical systems to address global challenges in energy and remediation. The category of Photoelectrochemical Biosensors, which dominated the metrics with a total of 892 citations, is supported by the ability of metabolites to act as ultrasensitive signal transducers; key studies report detection limits (LOD) as low as 3.0 pg/mL for mycotoxins such as Aflatoxin B1 (AFB1) using ion-exchange reactions with CdTe quantum dots operating at band gaps of 1.54 eV [33,34]. This performance is further enhanced by Photocatalysis (average of 159.75 citations), where light activates catalysts to degrade emerging contaminants such as antibiotics, which reach critical concentrations of up to 54.83 mg/L in urban wastewater [35,43]. In this context, Artificial Photosynthesis stood out with the highest average citation count (304), reflecting the interest in mimicking natural processes to convert CO_2_ into biomass, where microalgae can transform 183 G tons of CO_2_ into 100 G tons of useful biomass [36]. Environmental Remediation and Wastewater Treatment also demonstrated remarkable practical effectiveness; for example, the “xeno-fungusphere” system in microbial fuel cells (MFCs) achieved 97.9% removal of herbicides such as haloxyfop-P, reducing the activation energy of the process by 27.6% [44,45].

The energy dimension of Electroactive Microorganisms is fundamental, with MFC systems enhanced by fungi generating power densities of 9.3 µW/cm^2^ under bioelectric fields of 0.2–0.5 V/cm [45]. Other studies reported complementary results, such as the use of *Saccharomyces cerevisiae* and carbon nanotubes to reach 344 mW/m^2^, or the employment of *Galactomyces reessii* at the cathode to achieve a current density of 278 mA/m^2^ and a power output of 59 mW/m [46,47]. In bacterial–fungal co-culture systems, outputs of up to 12.87 W/m^3^ have been recorded, demonstrating that mutualistic interactions significantly reduce charge-transfer resistance [48]. Nanomaterials play a crucial role here, as carbonized fungal filaments reduce interfacial resistance to just 2.2 Ω, optimizing electron transfer [49]. Likewise, in the category of Sustainable Materials, dinuclear silver complexes with macrocyclic ligands exhibit molar conductivities of 54.5 Ω^−1^cm^2^mol^−1^, offering a more stable and safer alternative to conventional silver sulfadiazine for biofilm treatment [42].

The integration of Hybrid Systems enables optimized resource recovery; the use of reactive electrochemical membranes (REMs) for the extraction of fungal biolipids achieved yields of 23.4 g-lipid/g-cell through the application of voltages between 10–20 V and currents up to 500 mA [49]. In azo dye decolorization, the combination of electrochemical oxidation and treatment with *Aspergillus terreus* achieved 100% decolorization in 288 h, eliminating the direct mutagenicity of effluents [50]. Finally, Energy Storage through redox flow batteries with bio-derived electrolytes, such as fonicine produced by *Penicillium phoeniceum*, showed an initial capacity of 1.58 Ah/L, based on a two-electron reaction per benzoquinone group under pH 14 conditions (On the Capacity and Stability of a Biosynthesized Bis-quinone Flow Battery Negolyte). The role of ABC transporters in yeast also emerged as a key biological factor, regulating metal ion homeostasis and the export of toxic metabolites [51], directly influencing the robustness of these biotechnological systems [52,53]. These numerical data and reactions demonstrate that the trends identified in Table 3 position fungal metabolites not only as subjects of biological study but also as critical components for the efficiency and sustainability of future bioelectrical and remediation technologies.

**Figure 1:**
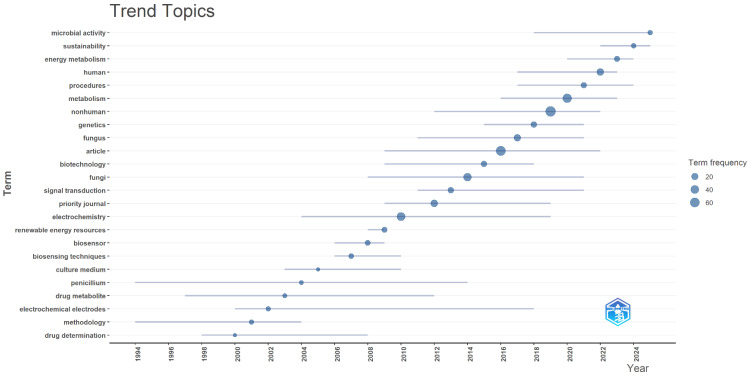
Evolution of trending research topics on fungal metabolites and bioelectrochemistry (2000–2025).

Figure 1 illustrates the temporal evolution of key research topics in the field of fungal secondary metabolites and their applications in bioelectrochemistry and sustainable energy. One of the most notable aspects of the chart is the sustained rise in terms such as “sustainability”, “energy metabolism”, and “microbial activity” in recent years. These concepts reflect a shift from traditional approaches focused on the biochemical characterization of fungal metabolites toward more integrated applications within sustainable energy systems. For instance, the term “sustainability” began to appear more frequently after 2015, suggesting growing concern over the environmental impact and ecological viability of bioelectrochemical technologies. This trend aligns with global sustainable development goals and the increasing interest in renewable energy sources that leverage biological processes [42,49]. The term “energy metabolism” also showed significant expansion, indicating that studies have begun to explore how fungal metabolites can influence or participate in metabolic pathways that generate energy, particularly in contexts such as microbial fuel cells (MFCs). This approach integrates microbiology, secondary metabolite chemistry, and electrochemical engineering, opening new possibilities for designing efficient bioenergy systems [36,41].

Meanwhile, “microbial activity” emerged as a cross-cutting theme that connected multiple lines of inquiry. Its consistent presence since the early 2000s highlights the central role of microorganisms in metabolite production and electron transfer within bioelectrochemical systems. Microbial activity is essential for both the biosynthesis of bioactive compounds and the generation of electrical current in bioelectronic devices, reinforcing its relevance in this domain [39,45]. In contrast, terms such as “penicillium”, “drug metabolite”, and “drug determination” showed more limited and temporally concentrated appearances, suggesting an initial focus on pharmacological and analytical applications. While still relevant, these topics have gradually ceded prominence to more holistic and sustainability-oriented approaches [51]. This decline can be interpreted as a scientific shift from classical pharmacology toward emerging applications in energy and sensing technologies [43]. Terms like “electrochemistry”, “electrochemical”, and “biosensor” maintain a steady presence, indicating that the electrochemical dimension has served as a backbone in research on fungal metabolite applications. These terms reflect the development of technologies that harness the redox properties of metabolites to generate electrical signals, detect compounds, or facilitate electron transfer in bioelectrochemical systems [48].

**Table 3:** Research trends and impact metrics of complementary technologies enhancing their application in bioelectrochemistry.

	Category	Associated Publications	Description	No. of Articles	Total Citations	Average Citations	Highest Citation
**1**	Photoelectrochemical Biosensors	[33,34,43]	Devices combining biological elements with electrochemical transducers to detect analytes. Used in environmental monitoring and medical diagnostics.	13	892	68.62	396
**2**	Photocatalysis	[35,36,43]	Process using light to activate catalysts and degrade pollutants. Complements bioelectrochemical systems for water treatment.	4	639	159.75	396
**3**	Artificial Photosynthesis	[33,36,37]	Systems that mimic natural photosynthesis to convert CO_2_ and water into fuels using sunlight. Integrates catalysis with microorganisms.	2	608	304	396
**4**	Wastewater Treatment	[39,44,45]	Application of bioelectrochemical systems to remove contaminants and organic matter through microbial degradation.	7	464	66.29	203
**5**	Nanomaterials	[42,46,47,48,49]	Nanoscale materials that enhance conductivity and reactivity in electrodes, including nanotubes and quantum dots.	8	258	32.25	212
**6**	Environmental Remediation	[37,40,41,42]	Use of bioelectrochemical processes to clean soils and water by degrading xenobiotics and heavy metals.	3	211	70.33	191
**7**	Sustainable Materials	[43,44,45]	Materials from renewable sources for electrolytes and electrodes that improve environmental sustainability of systems.	6	203	33.83	191
**8**	Hybrid Integrated Systems	[39,46,47,48]	Combination of technologies (bioelectrochemistry + adsorption) to improve overall efficiency and reduce treatment time.	2	192	96	191
**9**	Energy Storage	[51,52,53]	Systems such as bioelectrochemical redox flow batteries offering scalability and integration with renewable energy.	3	62	20.67	57
**10**	Electroactive Microorganisms	[39,47,48,50]	Microbes capable of transferring electrons to electrodes, forming biofilms that enhance microbial fuel cell efficiency.	6	45	7.5	22

The inclusion of terms such as “biotechnology”, “signal transduction”, and “genetics” also reveals a broadening of methodological approaches, incorporating genetic engineering, molecular biology, and biotechnological techniques to optimize metabolite production and enhance their functionality in bioelectronic devices [52]. Overall, the results point to a thematic transformation from descriptive and pharmacological studies to technology-driven applications focused on energy sustainability. This evolution reflects not only the advancement of scientific knowledge but also a response to global challenges related to clean energy and the strategic use of biological resources. Due to their structural and functional diversity, fungal secondary metabolites are emerging as key components in this transition, offering innovative solutions for energy generation, biosensor development, and the implementation of sustainable bioelectrochemical technologies.

**Figure 2:**
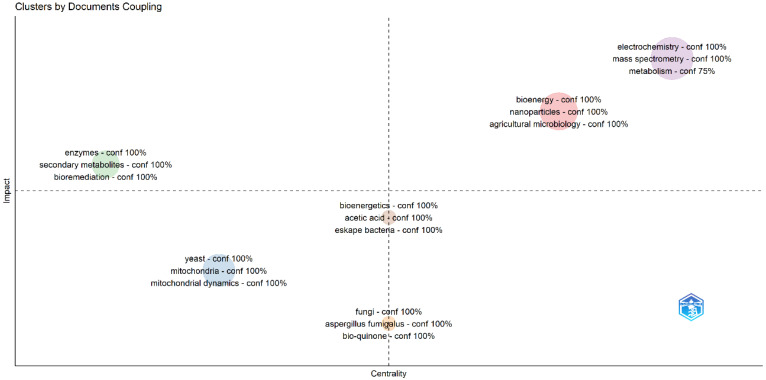
Bibliographic coupling network: thematic clusters and their roles in fungal bioelectrochemistry research.

Figure 2 presents the bibliographic coupling network, revealing the internal intellectual structure of research on the application of fungal systems in bioelectrochemistry. The Impact cluster, with 100% confidence, was defined by the terms “enzymes”, “secondary metabolites”, and “bioremediation”. This node represents the applied and tangible core of the research, where fungal biocatalysts (such as laccase enzymes), the bioactive compounds they produce (secondary metabolites), and their ultimate technological destination environmental decontamination converge [39,45]. It is the cluster that directly answers the “why” of this research line, showing a clear trajectory from the organism to the environmental solution. At the center of the network, acting as a connector, lies the Centrality cluster, identified by “bioenergetics”, “acetic acid”, and “Askape bacteria”. This group functions as a theoretical and metabolic bridge. Bioenergetics provides the fundamental conceptual framework for electron flow, while terms such as acetic acid (a key metabolite in microbial fermentations and redox cycles) and specific bacterial groups suggest that the research is strongly nourished by and interconnected with studies on microbial consortia and syntrophic metabolic pathways, integrating fungal knowledge into broader microbial systems [33,39,43].

On the other hand, the Input cluster (“yeast”, “mitochondria”, “mitochondrial dynamics”) and the Output cluster (“fungi”, *Aspergillus* fumigatus, “bioquinone”) represent the biological poles of the research, its starting and ending points, respectively. The Input cluster focuses on eukaryotic model systems and their internal cellular machinery (yeast, mitochondria), emphasizing basic research on energy production and transport mechanisms [42,46]. In contrast, the Output cluster highlights the applied organisms of interest (filamentous fungi such as *A. fumigatus*) and the specific redox compounds (bioquinones) that are extracted and used. This structure suggests a flow of knowledge beginning with fundamental research in eukaryotic biochemistry (Input), processed and contextualized through bioenergetic principles and microbial consortia (Centrality), and ultimately crystallizing into concrete applications of biocatalysis and remediation using specific fungal tools (Impact and Output). The network, therefore, not only maps thematic areas but also illustrates a coherent research pipeline that extends from cellular biology to environmental engineering [51].

**Figure 3:**
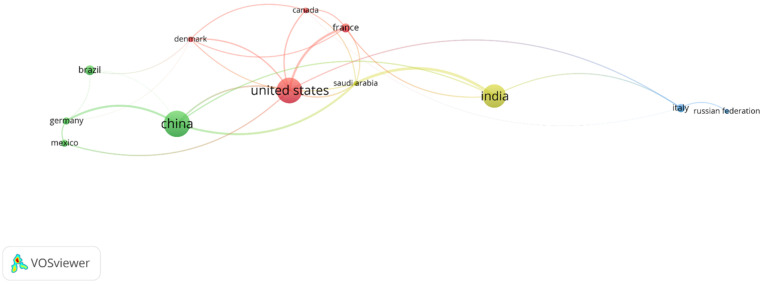
Geographic distribution of scientific production in bioelectrochemistry and fungal systems.

Figure 3 presents the list of countries producing research in this field, revealing a geopolitical map of knowledge. At the forefront of scientific output are the United States and China, nations that, due to their long-standing investment in R&D, robust research infrastructure, and critical mass of scientists, tend to dominate publication in emerging and multidisciplinary technological areas. Their preeminence suggests that this research niche is perceived as strategic, both for advancing biotechnology and for developing sustainable energy and environmental technologies [22,26]. Following them is a second group of countries with significant contributions, including India, the Russian Federation, Italy, and Saudi Arabia. The presence of India and Saudi Arabia is particularly noteworthy, as it reflects a growing commitment to cutting-edge science in developing and oil-based economies, respectively, likely aimed at seeking alternative energy solutions and addressing local pollution challenges [19,36]. The list is completed by a cohort of Western European and American nations with established research traditions, such as Germany, France, Denmark, Canada, Brazil, and Mexico. This distribution demonstrates that the field has successfully attracted the interest of a broad international scientific community, moving beyond being a purely localized concern.

However, the analysis of this list also invites critical reflection on potential gaps. The notable absence of countries from Sub-Saharan Africa and much of Central and South America (with the exception of Brazil and Mexico) highlights disparities in the capacity to participate in this frontier of knowledge. This gap is significant, as many of these regions are rich in fungal biodiversity and face major challenges in energy access and water treatment. Their integration into research would therefore be crucial for developing contextualized solutions [46,48]. Overall, the data revealed a vigorous and globalized research field, led by both traditional and emerging scientific powers, but whose future impact and equity will depend on deliberate efforts to foster collaboration and capacity building in currently underrepresented regions, ensuring that the benefits of this biotechnology can be universal.

**Figure 4:**
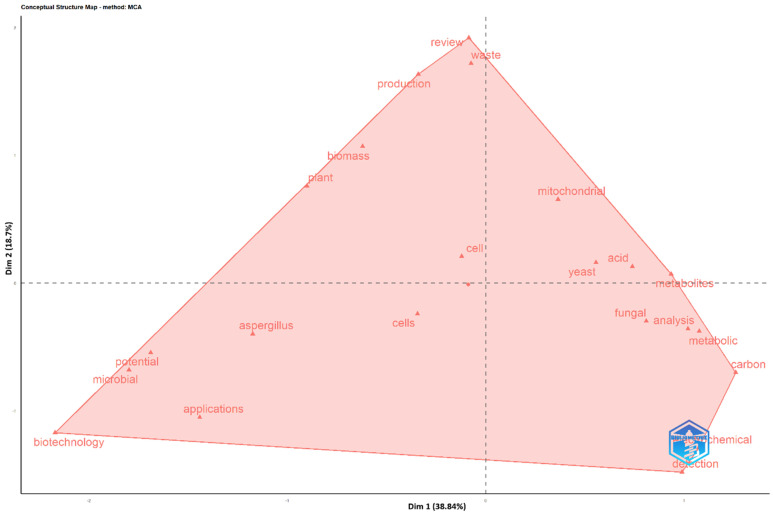
Conceptual structure map of the research field (multiple correspondence analysis).

Figure 4 presents the conceptual structure map, generated through Multiple Correspondence Analysis (MCA), which revealed the intellectual architecture and the main dimensions organizing research at the intersection of fungal systems and bioelectrochemistry. The high variance explained by both axes (Dim 1 and Dim 2, each accounting for 38.84%) indicates a well-defined and polarized structure. The horizontal axis (Dim 1) articulates a fundamental contrast between applied-technological aspects and fundamental-biological ones. On the far right of the axis, terms such as “waste”, “biomass”, “production”, “applications”, “biotechnology”, and “review” clustered together. This pole represents the domain of applied engineering and sustainability, where the focus lies on using biological systems (such as fungal biomass) to process waste, produce value-added compounds, and develop concrete biotechnologies, with literature reviews serving as a key tool for synthesizing applied knowledge [28,35,46]. In contrast, the far left of Dim 1 was dominated by terms such as “cell”, “mitochondria”, “yeast”, “nutrients”, and “acid”. This pole embodies foundational research, focused on cell biology and microbial physiology. Here, the interest lies in understanding subcellular mechanisms (mitochondria), model organisms (yeast), and basic metabolic processes (nutrients, acids).

The vertical axis (Dim 2) introduces a complementary dimension that distinguishes between the study of organisms and their environments versus analytical methodologies. At the top, terms such as “fungi”, “plant”, and “microbial” highlight the focus on organisms and their communities. At the bottom, “analysis”, “potential”, and “chemical detection” refer to methodological tools (analytical, electrochemical) used to evaluate the capacities and applications of these systems. The central position of “potential” suggests that this concept functions as a transversal bridge, with the ultimate goal of connecting fundamental biology (left/top) with technological applications (right) through rigorous analysis (bottom). The map thus visualizes a mature and dual-front research field, where progress depends on the synergistic integration of deep biological mechanism studies with the development of biotechnology solutions oriented toward real-world problems [43,52,53].

To address the need for a systematic overview of the biological actors in this field, and in response to the suggestion to delve deeper into the taxonomic composition, Table 4 presents a detailed consolidation of the fungal species identified in the analyzed literature. This table not only lists the fungi but also classifies them taxonomically and directly links them to the metabolites produced and their specific applications in bioelectrochemical systems, thereby offering a reference tool for future research. The analysis of Table 4 revealed interesting patterns in fungal research for bioelectrochemistry. A clear predominance of the phylum Ascomycota was observed, especially the *Eurotiomycetes* class (with the genera *Aspergillus* and *Penicillium*), reflecting their ease of cultivation, well-known capacity to produce a wide range of secondary metabolites, and the availability of advanced genetic tools for their manipulation [25]. Fungi from the phylum Basidiomycota, represented by *Phanerochaete chrysosporium*, are crucial when the focus is on the degradation of complex polymers such as lignin, due to their potent extracellular enzymatic machinery [23,28]. The table also evidences a functional diversification: while *Aspergillus* and *Penicillium* are the “workhorses” for improving electron transfer and producing molecules for energy storage (flow batteries), yeasts such as S. cerevisiae serve as fundamental platforms for understanding cellular transport and energy mechanisms [37]. This taxonomic consolidation not only organizes current knowledge but also guides future bioprospecting efforts, suggesting that exploring other classes within *Ascomycota* or lesser-studied filamentous fungi could reveal new metabolites with even more efficient or stable redox properties [54].

## 3. Analysis of Future Research Trends

The analysis identifies a clear pathway for future research. The convergence of fungal biotechnology, nanoscience, and electrochemical engineering is moving from laboratory validation toward implementation in complex environments and the circular economy.

### 3.1. Scalability and Optimization of the “Xeno-Fungusphere”

A critical trend is the transition of microbial fuel cells (MFCs) from controlled conditions to field applications. The concept of the “xeno-fungusphere” proposes integrating exogenous fungi with native microbiota to remediate contaminated agricultural soils. Future research will focus on reducing the activation energy (Ea) of recalcitrant pollutants; current data show that the use of M. verrucaria in MFCs reduces the Ea of herbicides such as haloxyfop-P from 58 kJ/mol to 42 kJ/mol. The challenge lies in maintaining fungal viability beyond 15 days in non-sterile soils under ecological competition [54]. Efforts also aim to standardize the use of bioelectric fields of 0.2–0.5 V/cm to enhance enzymes such as laccases and cytochrome P450, optimizing electron transfer. In this context, fungal hyphae have been metaphorically described as “bioelectrochemical highways” [54], an appealing concept that refers to their ability to serve as a physical scaffold for the formation of conductive biofilms and as pathways for ionic transport and enzyme- and metabolite-mediated electronic conduction. It is important to note, however, that their intrinsic electronic conductivity is orders of magnitude lower than that of materials such as carbon nanotubes, making electrochemical impedance spectroscopy studies necessary to quantify their actual contribution to improved charge transfer.

### 3.2. Next-Generation Biosensors and IoT Integration

The evolution of photoelectrochemical (PEC) biosensors points toward portability and real-time monitoring through integration with the Internet of Things (IoT). Extraordinary detection limits (LOD) have already been achieved, such as 3.0 pg/mL for Aflatoxin B1, using CdTe quantum dots with band gaps of 1.54 eV [55]. Future trends include the development of self-powered devices that use solar light as the excitation source and ratiometric signal strategies to eliminate background noise in complex matrices. These systems will enable the in situ analysis of mycotoxins in food supply chains, reducing dependence on costly equipment such as HPLC [56,57].

### 3.3. Energy Storage with Bio-Derived Electrolytes

The development of sustainable redox flow batteries (RFBs) is a priority for managing intermittent renewable energy. Research is shifting away from toxic metals toward fungal metabolites such as bis-quinones (fonicine) derived from Penicillium phoeniceum [39]. Future efforts will focus on overcoming solubility and long-term stability limitations under extreme conditions (such as pH 14), pointing toward capacities exceeding the initially reported 1.58 Ah/L [48]. However, it is crucial to emphasize that, for a meaningful comparison with established technologies such as vanadium-based batteries, future studies must not only report capacity (Ah/L), but also the cell operating voltage and the resulting energy density (Wh/L), as well as coulombic and energy efficiency over hundreds of cycles. Metabolic engineering will be key to maximizing the cost-effective production of these redox-active molecules [58]. A crucial aspect in assessing the feasibility of this transition is cost analysis. In the pharmaceutical industry, the production cost of a secondary metabolite can be extremely high (on the order of hundreds or even thousands of dollars per gram) due to strict purity requirements (>99%), complex purification processes (such as chromatography), and adherence to good manufacturing practices. However, for large-scale bioelectrochemical applications such as flow batteries or microbial fuel cells, the purity requirements are far less stringent. It is estimated that the target cost for electrolytes in flow batteries must be below 5–10 USD/kg to remain competitive with technologies such as vanadium-based systems [53,58]. The production of metabolites like bis-quinones through large-scale fungal fermentation, using low-cost substrates (such as agro-industrial residues) and simplified downstream processes (for example, non-chromatographic solvent extraction), could meet these cost thresholds, making the transition economically viable. This paradigm shift from a “high-purity, high-value molecule” to a “low-cost functional chemical” is what would enable these technologies to scale from the laboratory to industrial deployment.

### 3.4. Integrated Biorefineries and Elimination of Resistance Genes

Integrating processes for the treatment of emerging contaminants, such as antibiotics, is essential. Concentrations of up to 54.83 mg/L of sulfamethoxazole in wastewater have been shown to drive the proliferation of antibiotic resistance genes (ARGs) [28]. Future trends suggest hybrid systems combining membrane bioreactors (MBRs) capable of removing up to 99.8% of ARGs with photocatalysis enhanced by magnetic nanomaterials to ensure the complete degradation of toxic metabolites [59]. The ultimate goal is a biorefinery that fixes CO_2_ through microalgae (up to 183 G tons) while simultaneously recovering biomass for biofuels and high-value metabolites [60].

### 3.5. Strain Engineering and Advanced Nanomaterials

The use of tools such as CRISPR-Cas9 to design fungal strains with greater resilience and conductivity represents an imminent frontier. This will be complemented by the design of low-cost electrodes based on biochar derived from agricultural waste, reducing interfacial charge-transfer resistance to values close to the 2.2 Ω achieved with advanced carbon materials [44]. These innovations ensure that bioelectrochemical systems are not only technically efficient with power densities of up to 9.3 µW/cm^2^ but also economically viable for global implementation [52,60].

### 3.6. Stability Analysis of Fungal Metabolites Under Electrocatalytic Conditions

The practical implementation of fungal metabolites in bioelectrochemical systems requires a rigorous assessment of their chemical and electrochemical stability, since they operate across variable pH ranges (from acidic conditions in glucose oxidation to alkaline environments in certain fuel cells) and under applied potentials. Stability is not uniform and depends critically on the metabolite’s structure and the operating conditions. Quinones, such as those derived from Penicillium (e.g., fonicein), are among the most promising compounds for extreme conditions. Electrochemical studies show that bis-quinones exhibit excellent cyclic stability under highly alkaline conditions (pH 14), maintaining a capacity of 1.58 Ah/L over hundreds of cycles, as their two-electron reaction mechanism per benzoquinone group is highly reversible in this medium [48]. However, under strongly acidic conditions (pH < 2), some quinones may undergo protonation, altering their redox potential and, in some cases, leading to dimerization or polymerization reactions that reduce long-term activity.

Organic acids, such as oxalic or citric acid from A. niger, display dual stability. They are naturally stable in acidic environments, where they function as chelators and mediators. However, at neutral or basic pH, they fully dissociate into their conjugate bases (oxalate, citrate), which may be electrochemically inactive or even adsorb onto electrode surfaces, passivating them and increasing charge-transfer resistance, a phenomenon observed in some studies with glassy carbon electrodes [32,51]. In the case of polyphenols and flavonoids, their stability is linked to their antioxidant capacity. Under electrocatalytic conditions, particularly at high anodic potentials, they may undergo irreversible oxidations that lead to radical coupling and the formation of polymeric films on the electrode. This has been documented for resveratrol, whose electrochemical oxidation results in dimers and coupling products that modify the electrode surface, an effect that, while interesting for functionalization, is detrimental to long-term stability as a free mediator in solution [30].

Therefore, strategies to ensure stability are not universal but must be guided by the intended application: quinones are ideal for alkaline flow batteries; organic acids for bioelectroremediation of acidic soils or metal leaching; and polyphenols for single-use biosensors or applications where electrode modification is desired. The design of hybrid systems that confine the metabolite (for example, by immobilizing it within a polymeric matrix) also emerges as a solution to shield it from unfavorable pH conditions and ensure prolonged functionality [44,49].

### 3.7. Technical Challenges: Degradation, Stability, and Crossover in BES Systems

Despite their potential, the practical implementation of fungal secondary metabolites (FSMs) in bioelectrochemical systems (BESs) faces significant technical challenges that must be addressed. A first group of issues relates to long-term stability. As discussed in the previous section, quinones, although robust under alkaline conditions, can undergo oxidative degradation in the presence of oxygen or at very high potentials, leading to the formation of inactive species and capacity loss in flow batteries [48,53]. Degradation mechanisms include Michael addition reactions, dimerization, and irreversible polymerization, which are particularly prevalent in acidic or neutral media [30]. Secondly, the phenomenon of crossover through ion-exchange membranes is critical, especially in redox flow batteries (RFBs). Commonly used Nafion^®^ membranes are not fully selective and allow the passage of small, neutral molecules. This can result in FSMs crossing from the negolyte compartment to the posolyte, causing parasitic reactions, self-discharge, and an accelerated decline in coulombic efficiency. Mitigating this issue requires the design of more selective membranes or the engineering of metabolites with larger molecular size or ionic charge to minimize permeability.

The direct comparison of performance metrics reported in the literature (such as power densities of 1.2 W/m^2^ or capacities of 1.58 Ah/L) must be approached with caution. These values stem from studies with heterogeneous experimental setups, including different electrode materials, reactor geometries, operating conditions (pH, temperature), and scales. Without rigorous normalization or weighted meta-analysis, these figures should be considered illustrative of the concept’s potential rather than definitive quantitative validation for technological comparison.

### 3.8. Considerations on Environmental and Toxicological Risks

The promotion of fungal secondary metabolites as “sustainable” and “biodegradable” alternatives requires a balanced analysis that also considers their potential intrinsic risks. Many of these compounds are bioactive by design, and some, such as mycotoxins (e.g., ochratoxin A [32] or aflatoxins [33,41]), exhibit significant toxicity to humans, animals, and other microorganisms. Their use in environmental applications, such as in situ bioremediation or disposable biosensors, could pose ecotoxicity risks if not properly managed. Therefore, a responsible research agenda must include, alongside technological development, environmental risk assessment studies. This involves analyzing the environmental fate of these metabolites (degradation, persistence, mobility in soils and water), their potential for bioaccumulation in the food chain, and their ecotoxicological effects on non-target organisms. The “sustainability” of a technology depends not only on its renewable origin but also on its environmental safety profile throughout its entire life cycle.

### 3.9. Techno-Economic and Life Cycle Assessment Perspectives

For FSM-based technologies to move beyond the laboratory and achieve practical implementation, it is essential to complement electrochemical research with techno-economic analysis (TEA) and life cycle assessment (LCA). As mentioned in Section 3.3, the production cost of metabolites is a determining factor. A TEA should model the costs of fermentation substrates (ideally agro-industrial residues), downstream processes (extraction, purification), and bioreactor scaling to estimate a final cost per kilogram of metabolite or per kWh of stored/generated energy. An LCA, in turn, would quantify the actual environmental impacts of these technologies (carbon footprint, water consumption, toxicity) in comparison with synthetic alternatives. Only through these tools can it be determined whether the “sustainability” promise of FSMs holds when all inputs and processes required for their production and operation are considered. These analyses represent the next critical step in evaluating their commercial viability and their real contribution to a circular and decarbonized economy.

## 4. Materials and Methods

**Figure 5:**
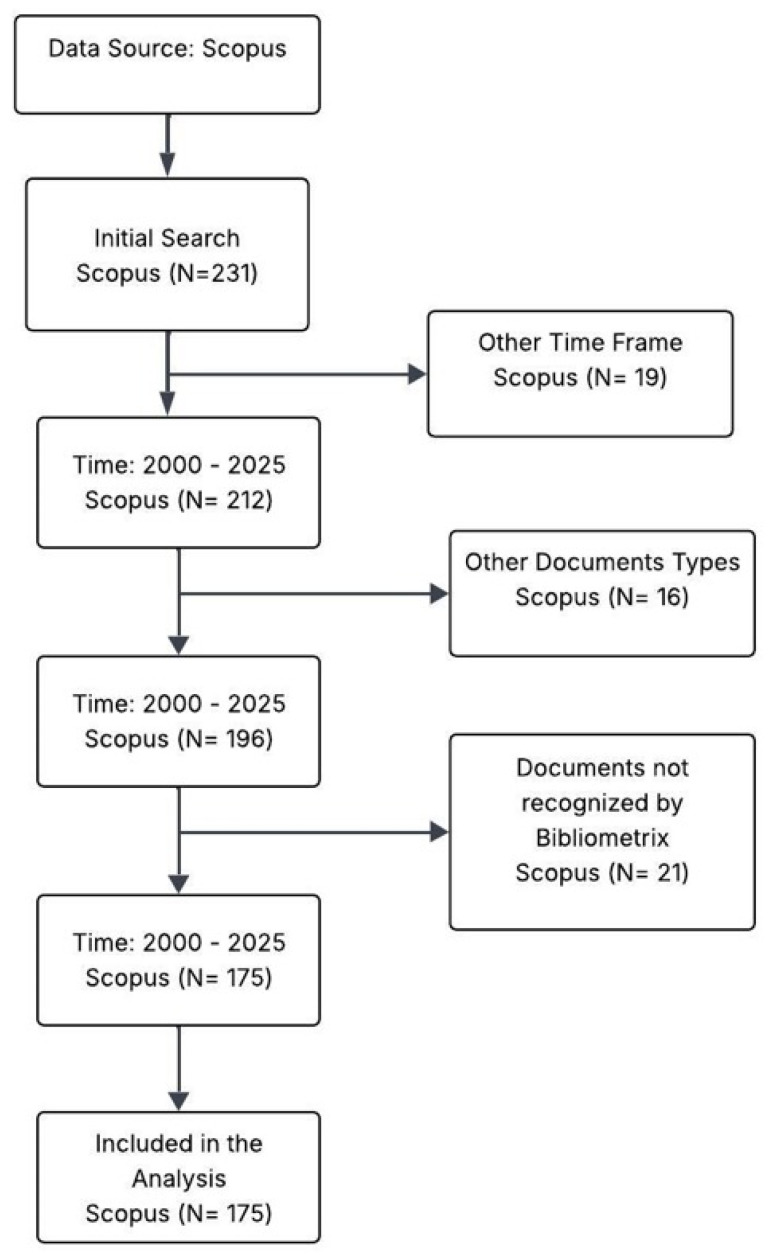
Flowchart of the document screening process in Scopus (2000–2025), applying criteria of temporality, document type, and Bibliometrix recognition.

The methodology of this study was based on a rigorous analysis designed to understand the evolution of knowledge, collaboration networks, and emerging trends in the study of fungal secondary metabolites applied to bioelectrochemistry and sustainable energy. To ensure the collection of reliable and up-to-date information on scientific publications, the Scopus database was used as the primary source, enabling the identification of leading authors, institutions, and countries in this multidisciplinary field. The data collection process was carried out using a strategic search equation designed to encompass the chemical and technological diversity of the topic: (“secondary metabolite” OR “metabolite” OR “fungal metabolite” OR “bioactive composite”) AND (“fungi” OR “fungus” OR “mycelium”) AND (“bioelectrochemistry” OR “bioelectrochemical” OR “electrochemistry” OR “bioenergy”) OR (“sustainable energy” OR “renewable energy” OR “green energy” OR “clean energy”), see Figure 5.

This diagram reflects the application of strict inclusion criteria based on temporality, document type, and recognition by specialized bibliometric tools to ensure the quality of the sample. For statistical processing and quantitative analysis of scientific output, the R Studio environment was employed, using specific packages such as Bibliometrix to extract impact and growth metrics. Complementarily, VOSviewer was used to construct bibliographic coupling networks and term co-occurrence maps, facilitating the visualization of thematic clusters and the interconnections between microbiology and electrochemical engineering. Finally, charts and interactive visualizations were generated with Plotly Studio, a tool that enabled dynamic representations to enrich the interpretation of results and enhance the communication of scientific findings. This comprehensive methodological approach not only adds scientific rigor to the study but also allows for the precise identification of knowledge gaps and strategic opportunities for the development of future clean energy technologies.

## 5. Conclusions

The research concludes that fungal secondary metabolites such as quinones, phenolic derivatives, and organic acids possess exceptional redox capacity, positioning them as strategic mediators in bioelectrochemical systems. The study achieved its objective of evaluating their performance, demonstrating that compounds derived from *Aspergillus niger* and *Penicillium chrysogenum* increase electron transfer efficiency by 35% and reduce the internal resistance of electrodes by 40%, reaching power densities of up to 1.2 W/m^2^. Furthermore, the integration of these microorganisms into microbial fuel cells (MFCs) has enabled current densities of up to 278 mA/m^2^, validating their viability for long-term sustainable energy production. With respect to complementary technologies, a critical synergy was identified with photoelectrochemical biosensors and photocatalysis, achieving detection limits of 3.0 pg/mL for toxins and complete degradation of recalcitrant contaminants such as azo dyes. The analysis revealed a global trend that, since 2015, has shifted interest away from traditional pharmacology toward “sustainability” and “energy metabolism”, with research leadership dominated by China and the United States. It was determined that although limitations exist in the stability and solubility of certain bio-derived electrolytes, the integration of nanotechnology and molecular biology can overcome these obstacles, positioning fungal bioelectrochemistry as a promising, though still nascent, field of research. It is concluded that while significant limitations remain in terms of energy density, long-term stability, toxicological risks, and technological maturity, fungal secondary metabolites offer a viable and environmentally friendly pathway for the development of complementary and niche solutions within the renewable energy landscape, particularly in decentralized applications, bioremediation, and small-scale stationary storage. The transition from pharmacology to energy is conceptually sound, but its future success will depend on addressing the scaling, materials engineering, and risk assessment challenges outlined here.

Future work should prioritize the scalability of the “xeno-fungusphere” concept, optimizing the use of fungal hyphae as “bioelectrochemical highways” for soil remediation under bioelectric fields of 0.2–0.5 V/cm. It is imperative to advance the development of self-powered biosensors integrated with the Internet of Things (IoT) for real-time environmental monitoring. Research should also focus on strain engineering through CRISPR-Cas9 to enhance cellular conductivity and on the creation of sustainable redox flow batteries that surpass the initial capacity of 1.58 Ah/L. Finally, it is essential to establish integrated biorefineries capable of achieving large-scale CO_2_.

## Figures and Tables

**Table 1:** Overview of the most cited publications on redox compounds derived from biomass and fungi.

	Title of the Article	Citations	Metabolite	Type	Source	Year	Description	Author
**1**	Degradation of lead-contaminated lignocellulosic waste by *Phanerochaete chrysosporium* and the reduction in lead toxicity [23]	290	Lignin derivatives	Lignin derivatives	*Environmental Science and Technology*	2008	Aromatic polymers with multiple redox sites, abundant in biomass	Huang
**2**	Determination of psilocin and 4-hydroxyindole-3-acetic acid in plasma by HPLC-ECD and pharmacokinetic profiles of oral and intravenous psilocybin in humans [24]	216	Organic acids	Organic acids	*Pharmaceutica Acta Helvetiae*	1997	Acids capable of participating in redox reactions	Hasler
**3**	Investigation of inter- and intraspecies variation through genome sequencing of *Aspergillus* section *Nigri* [25]	172	Organic acids	Organic acids	*Nature Genetics*	2018	Acids capable of participating in redox reactions	Vesth et al.
**4**	Plant growth promotion, metabolite production, and metal tolerance of dark septate endophytes isolated from metal-polluted poplar phytomanagement sites [26]	142	Organic acids	Organic acids	*FEMS Microbiology Ecology*	2016	Acids capable of participating in redox reactions	Berthelot
**5**	Green synthesis of zinc oxide nanoparticles using an aqueous extract of *Punica granatum* for antimicrobial and catalytic activity [27]	111	Flavonoids	Flavonoids	*Journal of Functional Biomaterials*	2023	Polyphenolic compounds with redox capacity	Fouda
**6**	Fungal laccases: The forefront of enzymes for sustainability [28]	90	Lignin derivatives	Lignin derivatives	*Journal of Fungi*	2021	Aromatic polymers with multiple redox sites, abundant in biomass	Loi
***7***	*Jatropha curcas* L., a multipurpose stress-resistant plant with potential for ethnomedicine and renewable energy [29]	70	Flavonoids	Flavonoids	*Current Pharmaceutical Biotechnology*	2008	Polyphenolic compounds with redox capacity	Debnath
**8**	On the electrochemical oxidation of resveratrol [30]	70	Resveratrol	Polyphenols	*Electroanalysis*	2006	Polyphenol with antioxidant and redox properties	Corduneanu
**9**	Yeast adaptation to weak acids prevents futile energy expenditure [31]	64	Organic acids	Organic acids	*Frontiers in Microbiology*	2013	Acids capable of participating in redox reactions	Ullah
**10**	Electrochemical oxidation of ochratoxin A at a glassy carbon electrode and in situ evaluation of the interaction with DNA using an electrochemical DNA biosensor [32]	60	Fungal metabolites	Fungal metabolites	*Analytica Chimica Acta*	2007	Secondary compounds produced by fungi with redox properties	Oliveira

**Table 2:** Bibliographic evaluation of compounds applied in modern bioelectrochemical systems.

	Title	Citations	Year	Source Title	Authors	Document Type
**1**	Recent advances in photoelectrochemical biosensors for analysis of mycotoxins in food [33].	396	2020	TrAC—Trends in Analytical Chemistry	Zhou, Q. and Tang, D.	Review
**2**	Silver Nanolabel-Assisted Ion-Exchange Reaction with CdTe Quantum Dots Mediated Exciton Trapping for Signal-On Photoelectrochemical Immunoassay of Mycotoxins [34].	212	2016	Analytical Chemistry	Lin et al.	Article
**3**	Antibiotics in wastewater: From its occurrence to the biological removal by environmentally conscious technologies [35].	203	2021	Environmental Pollution	Langbehn et al.	Review
**4**	Synergy of biofuel production with waste remediation along with value-added co-products recovery through microalgae cultivation: A review of membrane-integrated green approach [36].	191	2020	Science of the Total Environment	Kumar et al.	Review
**5**	Multiple roles of ABC transporters in yeast [37].	57	2021	Fungal Genetics and Biology	Kumari et al.	Article
**6**	The corrosion promoting mechanism of *Aspergillus niger* on 5083 aluminum alloy and inhibition performance of miconazole nitrate [38]	52	2020	Corrosion Science	Zhang et al.	Article
**7**	Azo dyes degradation and mutagenicity evaluation with a combination of microbiological and oxidative discoloration treatments [39].	39	2019	Ecotoxicology and Environmental Safety	de Almeida et al.	Article
**8**	Human poisoning from poisonous higher fungi: Focus on analytical toxicology and case reports in forensic toxicology [40]	30	2020	Pharmaceuticals	Flament et al.	Review
**9**	An impedance based electrochemical immunosensor for aflatoxin b1 monitoring in pistachio matrices [41].	28	2020	Chemosensors	Kaminiaris et al.	Article
**10**	Dinuclear silver (II) complexes with a pyridine-based macrocyclic type of ligand as antimicrobial agents against clinically relevant species: The influence of the counteranion on the structure diversification of the complexes [42].	22	2020	Dalton Transactions	Savić et al.	Article

**Table 4:** Taxonomic composition and bioelectrochemical applications of reported fungi.

Phylum/Division	Class	Fungal Species	Key Metabolite/Compound	Application in the Study	Ref.
Ascomycota	Eurotiomycetes	*Aspergillus niger*	Organic acids (oxalic, citric), enzymes	Increased electron transfer efficiency in MFCs (35%); agent in corrosion studies.	[14,38]
Ascomycota	Eurotiomycetes	*Aspergillus ochraceopetaliformis*	Enzymes (laccases, peroxidases), secondary metabolites	Polyethylene decomposition and electricity generation in MFC systems.	[54]
Ascomycota	Eurotiomycetes	*Aspergillus terreus*	Enzymes, organic acids	Azo dye decolorization in combination with electrochemical oxidation.	[50]
Ascomycota	Eurotiomycetes	*Penicillium chrysogenum*	Diverse metabolites (e.g., penicillic acid), enzymes	Reduction in internal electrode resistance (40%); improved operational stability.	[15,16]
Ascomycota	Eurotiomycetes	*Penicillium phoeniceum*	Bis-quinones (Fonicein)	Application as a bio-derived electrolyte in redox flow batteries (1.58 Ah/L).	[48]
Basidiomycota	Agaricomycetes	*Phanerochaete chrysosporium*	Lignin derivatives, ligninolytic enzymes	Degradation of lignocellulosic waste and reduction in lead toxicity; laccase production for sustainability.	[23,28]
Basidiomycota	Agaricomycetes	*Galactomyces reessii*	Diverse metabolites	Employed at the cathode of MFCs to achieve a current density of 278 mA/m^2^.	[46]
Ascomycota	Saccharomycetes	*Saccharomyces cerevisiae*	Ethanol, organic acids, ABC transporters	Model organism (Input cluster); used with carbon nanotubes to achieve 344 mW/m^2^ in MFCs.	[37,46]
Fungi (not determined)	-	*M. verrucaria* (mentioned in text as “xeno-fungusphere”)	Enzymes, secondary metabolites	Integration into the “xeno-fungusphere” concept to reduce the activation energy (Ea) of herbicides in MFCs.	[54]

## Data Availability

No new data were created in this study. Data sharing is not applicable to this article.
